# Vertebral artery stump syndrome after internal carotid artery stenting: A case report

**DOI:** 10.1097/MD.0000000000046628

**Published:** 2025-12-19

**Authors:** Subing Yin, Jun Zhu, Guoping Fu

**Affiliations:** a Department of Neurology, The Second Hospital of Shaoxing, Shaoxing, Zhejiang Province, China; b Department of Neurology, Huashan Hospital, Fudan University, Shanghai, China.

**Keywords:** black-blood magnetic resonance imaging, case report, internal carotid artery stenting, vertebral artery stump syndrome

## Abstract

**Rationale:**

Vertebral artery stump syndrome (VASS) is an uncommon etiology of posterior circulation ischemic stroke. Previous studies have proposed several possible pathogenic mechanisms. However, the precise pathogenesis and initiating factors remain unclear. We present a case of VASS occurring following carotid artery stenting, in which thrombus formation was identified in the vertebral and basilar arteries using black-blood magnetic resonance imaging (MRI). Based on these findings, we propose a plausible pathogenesis and potential triggering factors.

**Patient concerns:**

We report a case of posterior circulation ischemic stroke following carotid artery stenting, suggesting VASS as the etiology.

**Diagnoses:**

The diagnosis of VASS was made based on the presence of posterior circulation ischemic stroke, occlusion at the origin of the vertebral artery, antegrade flow in the distal segment of the ipsilateral vertebral artery, and the coexistence of an intracranial arterial occlusion distal to the extracranial lesion, after excluding other potential causes.

**Interventions:**

We employed a treatment strategy combining antiplatelet and anticoagulant therapy.

**Outcomes:**

Thrombi were directly visualized in the basilar and vertebral arteries on black-blood MRI, with consistent signal characteristics. The patient’s symptoms demonstrated improvement following the implemented treatment regimen.

**Lessons:**

Based on this case, we recommend a comprehensive black-blood MRI evaluation for potential VASS in patients with vertebral artery occlusion prior to performing carotid artery stenting.

## 1. Introduction

Ischemic stroke occurring in the region supplied by an occluded carotid artery is termed carotid stump syndrome. Similarly, acute ischemic stroke in the territory affected by vertebral artery occlusion is known as vertebral artery stump syndrome (VASS), first described by Nguyen in 2008.^[1]^ Although several case reports have since been published, we present a case of VASS occurring after carotid artery stenting, where thrombi were directly observed in the vertebral artery stump and basilar artery on black-blood Magnetic resonance imaging (MRI) – a finding not previously described in the literature.

## 2. Case presentation

**Figure 1.**
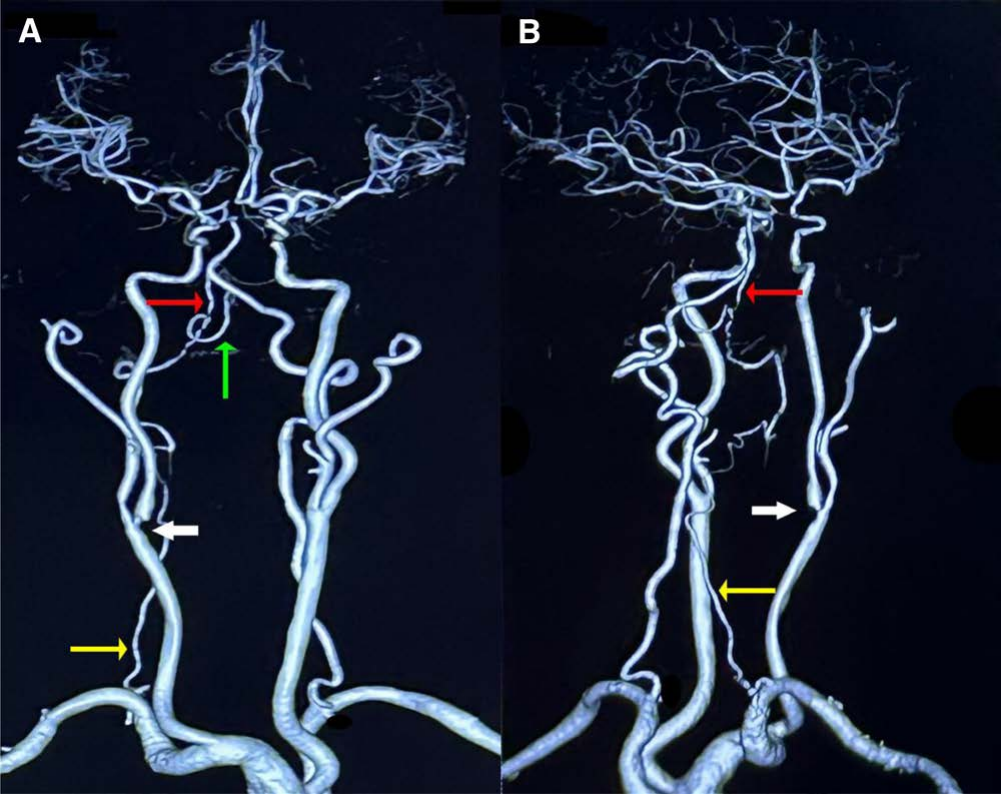
(A and B) The CTA revealed severe stenosis at the origin of the right internal carotid artery (white arrow) and occlusion of the right vertebral artery. The right deep cervical artery (yellow arrow) was connected to the V2 segment of the right vertebral artery (red arrow), and a prominent right PICA was observed (green arrow). The left vertebral artery and the left internal carotid artery appeared normal. CTA = computed tomography angiography, PICA = posterior inferior cerebellar artery.

**Figure 2.**
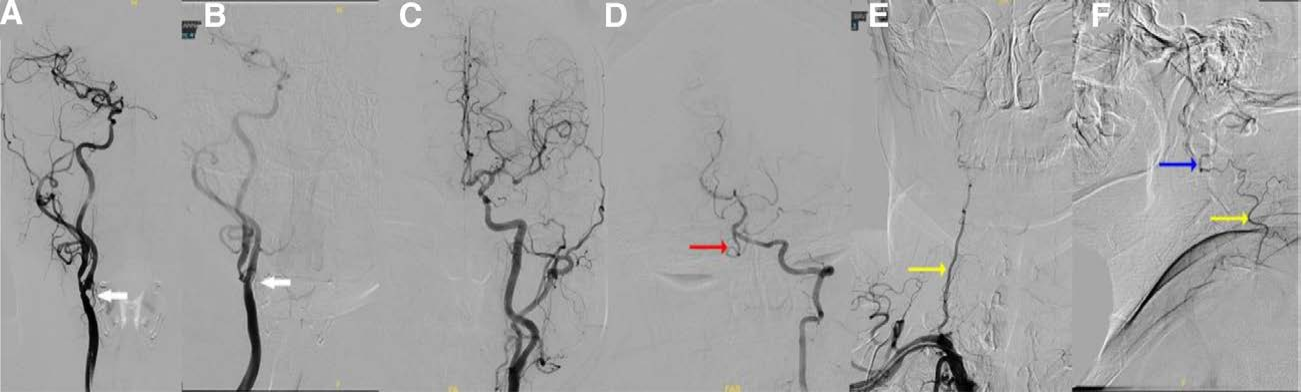
(A) Severe narrowing of the right internal carotid artery (white arrow). (B) Postimplantation surgery (white arrow). (C) Left internal carotid artery. (D) Left vertebral artery imaging shows simultaneous visualization of the right vertebral artery V4 (red arrow). (E and F) The right deep cervical artery (yellow arrow) is connected to the right vertebral artery V2 (blue arrow).

**Figure 3.**
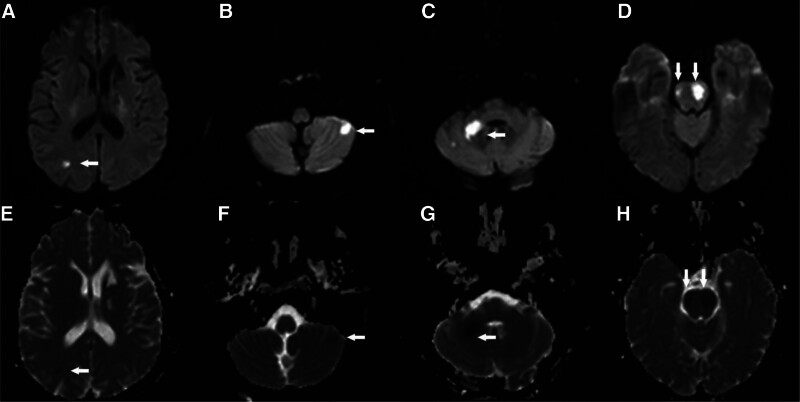
(A–D) Acute cerebral infarction on DWI sequence (white arrow). (E–H) Low signal on the corresponding ADC sequence (white arrow). ADC = apparent diffusion coefficient.

**Figure 4.**
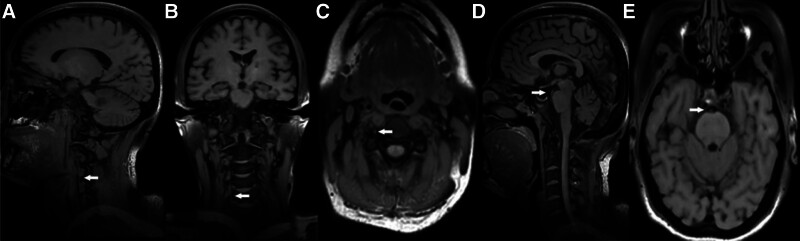
(A–C) The black-blood sequence shows a thrombus within the right vertebral artery in sagittal, coronal, and horizontal orientations (white arrow). (D–E) The black-blood sequence shows a thrombus within the basilar artery in sagittal and horizontal orientations (white arrow).

A 46-year-old Chinese woman with a history of hypertension and type 2 diabetes was referred to our hospital after computed tomography angiography revealed severe stenosis of the right internal carotid artery (Fig. 1A and B). Subsequent DSA demonstrated approximately 80% stenosis of the right internal carotid artery and complete occlusion at the origin of the right vertebral artery (Fig. 2A). Notably, blood flow was observed to redirect from the deep cervical artery to the distal end of the V2 segment via anastomosis (Fig. 2E and F). Following stent placement in the right internal carotid artery, the patient presented with somnolence, aphasia, and dysphagia, with muscle strength graded 3 in the left limbs and 4 in the right limbs, along with bilateral positive Babinski signs. the patient exhibited an acute cerebral infarction impacting the occipital lobe, cerebellum, and brainstem. (Fig. 3A–H). Thrombi were discerned within the occluded right vertebral artery and basilar artery via black-blood MRI (Fig. 4A–E). Our patient presented with acute posterior circulation stroke. Both computed tomography angiography and DSA confirmed occlusion at the origin of the vertebral artery, with antegrade flow in the distal segment of the ipsilateral vertebral artery, as well as the coexistence of an occluded intracranial artery distal to the extracranial occlusion. Given that the left vertebral and basilar arteries were normal, postoperative blood pressure was stable, and 24-hour Holter electrocardiogram (ECG), echocardiography, D-dimer, FVIII, and antiphospholipid antibody tests showed no abnormalities, we considered this case to meet the VASS criteria proposed by Zhang et al.^[2]^ The patient declined endovascular intervention. For treatment, the patient received aspirin enteric-coated tablets (100 mg qd) in combination with clopidogrel (75 mg qd) for 17 days prior to the procedure. Postoperatively, the regimen was adjusted to aspirin (100 mg qd) plus nadroparin (2050 IU subcutaneously q12h) for 14 days. Once the patient’s condition stabilized, therapy was switched to aspirin (100 mg qd) combined with clopidogrel (75 mg qd) for 6 months, followed by aspirin monotherapy (100 mg qd). The patient tolerated the medications well, with no recurrence of ischemic stroke or hemorrhagic events. At the 1-year follow-up, the patient’s swallowing function had essentially recovered to normal. However, dysarthria persisted, though she was able to ambulate independently. We recommend a black-blood MRI for potential VASS in patients with vertebral artery occlusion before carotid artery stenting.

## 3. Discussion

VASS, as one of the causes of posterior circulation ischemic stroke, is often overlooked. It accounts for approximately 1.4% of posterior circulation ischemic stroke cases and about 8% of acute basilar artery occlusions.^[3,4]^ Due to its relatively high recurrence rate 25% and poor prognosis 25%, it is crucial to recognize and address this condition.^[3]^ Nguyen initially proposed 3 potential mechanisms of onset, but the precise pathogenesis of VASS remains unclear.^[1]^ Tempaku directly observed blood flow stasis with to-and-fro flow in the vertebral artery via DSA.^[5]^ Suzuki detected turbulent flow at the occlusion site of the vertebral artery using ultrasound.^[6]^ Ji confirmed a filling defect at the origin of the left vertebral artery, consistent with thrombus, through pathological examination.^[7]^ Additionally, we directly visualized thrombus at the vertebral artery stump using black-blood MRI. The similarity of intraluminal thrombus signal characteristics between the basilar artery and the proximal vertebral artery on black-blood MRI suggests a shared origin. This observation supports the hypothesis that the thrombus within the basilar artery likely resulted from the migration of an older thrombus from the vertebral artery. Therefore, we propose that the mechanism of VASS may involve thrombus formation at the vertebral artery stump due to turbulence and/or low blood flow, which is subsequently transported to the intracranial circulation through turbulence and/or collateral pathways. Currently, there is no literature addressing the initiating factors of ischemic stroke in VASS. Given that our patient experienced an ischemic stroke following carotid artery stenting, we suspect that heparin used during and after the procedure may be a contributing factor. However, this hypothesis requires further investigation for confirmation. One limitation of our study is that endovascular thrombectomy and recanalization were not performed. Pathological verification that the thrombi within the basilar and vertebral arteries originated from the same source would further substantiate the proposed mechanism of VASS. The major challenge was selecting an appropriate treatment after the patient declined endovascular therapy. Although we chose combined anticoagulant and antiplatelet therapy, considering that artery-to-artery embolism may underlie its pathogenesis, we believe that this treatment strategy requires further investigation. This is because anticoagulation might act as a triggering factor for VASS and could potentially lead to recurrent ischemic stroke. Although extensive research has been conducted on the treatment of VASS, the optimal therapeutic approach remains undefined. Given its pathogenesis, endovascular treatment to remove the vertebral artery stump appears to be the preferred approach, as this intervention offers a high success rate for recanalization.^[8]^ Although a case report has shown no recurrence after 7 years of antiplatelet therapy, we believe that VASS represents an artery-to-artery embolism.^[9]^ In this context, anticoagulant therapy may be the preferred first-line treatment, particularly if endovascular intervention is not performed.^[10]^ Posterior circulation infarction during carotid artery stenting is rare. In addition to VASS, other potential mechanisms should be considered. One is artery-to-artery embolism via anomalous channels between the anterior and posterior circulations, such as internal carotid–vertebral anastomoses (persistent trigeminal, hypoglossal, proatlantal intersegmental, or otic arteries) or external carotid–vertebral anastomoses (via the occipital or ascending pharyngeal artery), which can be identified on DSA. Procedure-related factors, including dissection at the origin of the deep cervical artery or inadvertent guidewire insertion into an occluded vertebral artery, may also contribute and can be detected by DSA. Additionally, perioperative conditions that mimic posterior circulation infarction should be distinguished, including cerebral hyperperfusion syndrome (characterized by elevated intracranial flow velocity, malignant hypertension, and brain edema), contrast-induced encephalopathy (showing DWI hyperintensity without corresponding apparent diffusion coefficient changes and and cortical or subarachnoid hyperdensities on CT), and reversible posterior leukoencephalopathy syndrome (featuring headache and symmetric bilateral posterior vasogenic edema on MRI).^[11–14]^ VASS, as a potential cause of posterior circulation ischemic stroke, warrants careful attention. Even prior to interventions in the anterior circulation, the possibility of VASS should be assessed, with black-blood MRI serving as a valuable tool to evaluate thrombus in the vertebral artery stump. If vertebral artery occlusion is detected preoperatively, performing endovascular treatment of the occluded vertebral artery may help prevent the development of VASS.^[8,15,16]^

## Acknowledgments

I sincerely thank Jianying Chen for her support, which allowed me to focus and successfully complete this paper.

## Author contributions

**Conceptualization:** Subing Yin.

**Funding acquisition:** Subing Yin.

**Resources:** Jun Zhu.

**Writing – original draft:** Subing Yin, Jun Zhu.

**Writing – review & editing:** Guoping Fu.
